# Dual “mAb” HER family blockade in head and neck cancer human cell lines combined with photon therapy

**DOI:** 10.1038/s41598-017-12367-7

**Published:** 2017-09-22

**Authors:** Jean-Baptiste Guy, Benoîte Méry, Edouard Ollier, Sophie Espenel, Alexis Vallard, Anne-Sophie Wozny, Stéphanie Simonet, Alexandra Lauret, Priscillia Battiston-Montagne, Dominique Ardail, Gersende Alphonse, Chloé Rancoule, Claire Rodriguez-Lafrasse, Nicolas Magné

**Affiliations:** 1 Université Lyon 1, UMR CNRS 5822 /IN2P3, IPNL, PRISME, Laboratoire de Radiobiologie Cellulaire et Moléculaire, Faculté de Médecine Lyon-Sud, F-69921 Oullins cedex, France; 2 Département de Radiothérapie, Institut de Cancérologie de la Loire - Lucien Neuwirth, 42270 Saint Priest en Jarez, France; 3 Département d’Oncologie Médicale, Institut de Cancérologie de la Loire - Lucien Neuwirth, 42270 Saint Priest en Jarez, France; 4 Dysfonction Vasculaire et Hémostase, INSERM, U1059, Saint-Etienne, France; 5 Hospices Civils de Lyon, Centre Hospitalier Lyon-Sud, 69495 Pierre-Bénite, France

## Abstract

Head and neck cancer stem cells (CSCs) are highly resistant to treatment. When EGFR is overexpressed in head and neck squamous cell carcinoma (HNSCC), HER2 and HER3 are also expressed. The aim of the present study was to investigate the effect of HER1/2/3 blockade through a combination of cetuximab and pertuzumab, with or without photon irradiation, on the proliferation and migration/invasion capabilities of an HNSCC chemo- and radioresistant human cell line (SQ20B) and its corresponding stem cell subpopulation. Cell proliferation, migration and invasion were studied after treatment with cetuximab +/− pertuzumab +/− 10 Gy photon irradiation. EGFR, phospho-EGFR, HER2 and HER3 protein expression levels were studied. Activation or inhibition of the RAS/MAPK and AKT-mTOR downstream signalling cascades was investigated through phospho-AKT and phospho-MEK1/2 expression. Cetuximab strongly inhibited SQ20B and FaDu cell proliferation, migration and invasion, whereas it had little effect on SQ20B-CSCs. Cetuximab–pertuzumab combined with radiation significantly inhibited SQ20B and FaDu cell and SQ20B-CSC proliferation, migration and invasion. Cetuximab–pertuzumab with 10 Gy photon irradiation switched off both phospho-AKT and phospho-MEK1/2 expression in the three populations. The triple therapy is therefore thought to inhibit SQ20B cells, SQ20B-CSCs and FaDu cells through an AKT-mTOR and Ras-MAPK downstream signalling blockade.

## Introduction

Head and neck squamous cell carcinoma (HNSCC) still has a dismal prognosis, despite recent biological and technological improvements^1^. In the past few years, it has been shown that the epidermal growth factor receptor (EGFR) is overexpressed in more than 90% of HNSCCs^2^. Faced with this therapeutic target, cetuximab, a mouse–human chimeric monoclonal antibody directed against EGFR, was developed and shown to significantly improve locoregional control, progression-free survival and overall survival when used concomitantly with radiotherapy (RT)^3,4^. These improvements were nevertheless counterbalanced by high rates of local and distant recurrences^4,5^ leading to specific mortality in the short or medium term^6^. The epithelial-to-mesenchymal process, giving invasion/migration capacities to cancer cells, is thought to be the root of all these recurrences. Furthermore, the presence of a subpopulation of cancer cells showing particularly high migratory potential^7^, known as cancer stem cells (CSCs), has been revealed in HNSCC^8^. Moncharmont *et al*.^9^ showed that a HNSCC CSC subpopulation with low EGFR expression could resist cetuximab, giving the first biological explanation for clinical reports. Moreover, increasing evidence suggests that cancers that initially respond to EGFR may subsequently become refractory, with a central role being played by human epidermal growth factor receptor (HER) family members (HER2 or erbB2, HER3 or erbB3, HER4 or erbB4)^10^. These receptors activate downstream signalling cascades including MAPK and PI3K/AKT through ligand-dependent homo- or heterodimerization, and thus enhance cell proliferation, angiogenesis, invasion and metastasis. Pertuzumab is an HER2 antagonist directed against the extracellular dimerization domain (subdomain II), and which blocks the ligand-dependent heterodimerization of HER2 with other HER family members, including EGFR or HER1, HER3 and HER4. As a result, pertuzumab inhibits ligand-initiated intracellular signalling through MAPK and PI3K/AKT. Interestingly, the HER2 and HER3 receptors are often expressed in HNSCC, making them potential new therapeutic targets^11,12^. Many ongoing studies are using pan-HER inhibitors in HNSCC, focusing on pan-HER tyrosine kinase inhibitors (clinicaltrials.gov: NCT02216916; Molecule: HM781-36B irreversible pan-HER inhibitor). However, a monoclonal coupled antibody blockade should enhance therapeutic efficacy and limit resistance with upstream and extracellular action^13^. Very few data exist on the HER2–HER3 blockade and invasion/migration in HNSCC. The combination of cetuximab with pertuzumab could block HER1, 2 and 3 and therefore target the major downstream signalling cascade of migration, proliferation and survival. The aim of the present study was to explore the effect of a pan-HER blockade on the proliferation, migration and invasion of human HNSCC cells and their corresponding CSC subpopulation, through combined treatment with cetuximab and pertuzumab, with or without photon irradiation.

## Results

### Basal cellular characteristics

#### HER family expression

**Figure 1:**
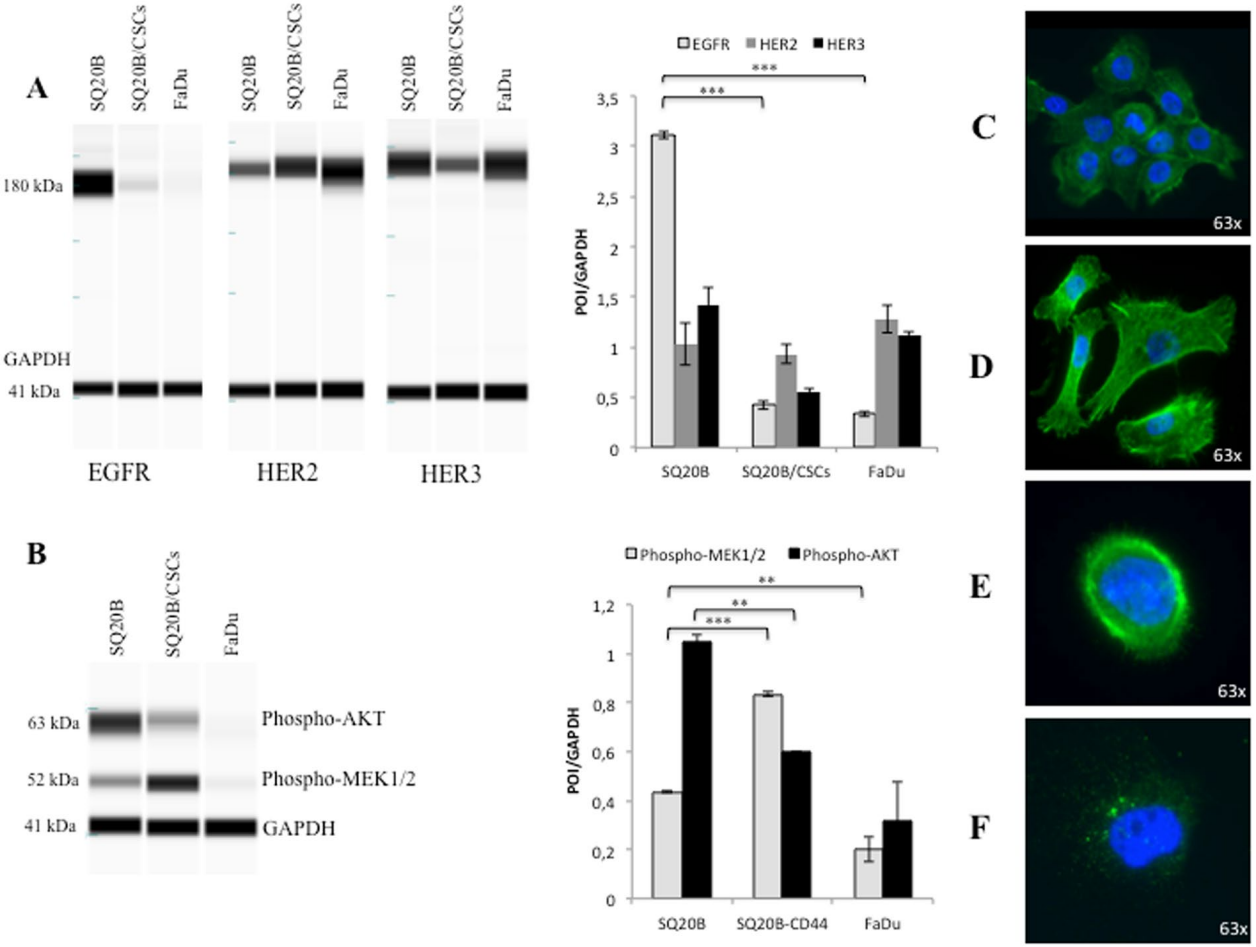
*Basal characteristics of SQ20B cells*, *SQ20B-CSCs and FaDu cells*. (**A**) EGFR (180 kDa), HER2 (184 kDa) and HER3 (189 kDa) basal expression in SQ20B cells, SQ20B-CSCs and FaDu cells. Protein expression was analysed with WES™ (a simple western blotting technique using an automated capillary-based size sorting system) and expressed graphically as the GAPDH ratio. (**B**) Phospho-AKT (Ser473) (63 kDa) and phospho-MEK1/2 (Ser217/221) (52 kDa) basal expression in SQ20B cells, SQ20B-CSCs and FaDu cells. Protein expression was analysed with WES™, and expressed graphically as the GAPDH ratio (protein-of-interest (POI)/GAPDH). (**C**) Microscopic observation (63×) of SQ20B cells stained with phalloidin (green actin) and DAPI (blue nucleus). (**D**) Microscopic observation (63×) of SQ20B-CSCs stained with phalloidin (green actin) and DAPI (blue nucleus). (**E**) Microscopic observation (63×) of a SQ20B cell stained with phospho-EGFR-FITC (green) and DAPI (blue nucleus). (**F**) Microscopic observation (63×) of a SQ20B-CSC stained with phospho-EGFR-FITC (green vacuoles) and DAPI (blue nucleus). Statistically significant differences are expressed as *p < 0.05, **p < 0.01 and ***p < 0.001. Each experiment was performed in triplicate.

EGFR was highly overexpressed in SQ20B cells, however the opposite occurred in SQ20B-CSCs and FaDu cells, in which EGFR was expressed at much lower levels (*P* < 0.001). HER2 and HER3 were expressed at almost equal levels in the three populations. HER2 expression was twice as high as that of EGFR in SQ20B-CSCs and FaDu cells (Fig. 1A).

#### Downstream signaling

Phospho-AKT expression was twice as high in SQ20B cells as in SQ20B-CSCs (*P* = 0.0018). By contrast, Phospho-MEK1/2 expression in SQ20B cells was half that in SQ20B-CSCs (*P* < 0.001). Phospho-AKT and Phospho-MEK1/2 (*P* = 0.003) were expressed less in FaDu cells (Fig. 1B).

#### Phenotypic characteristics

SQ20B cells displayed an epithelial phenotype, with many cell–cell junctions (Fig. 1C), whereas SQ20B-CSCs were organized in a mesenchymal phenotype with a tapered and sharp actin network (Fig. 1D). Under basal conditions, EGFR was localized on the cell membranes of SQ20B cells (Fig. 1E), and mostly within intracellular vacuoles in SQ20B-CSCs (Fig. 1F).

### Cell proliferation

**Figure 2:**
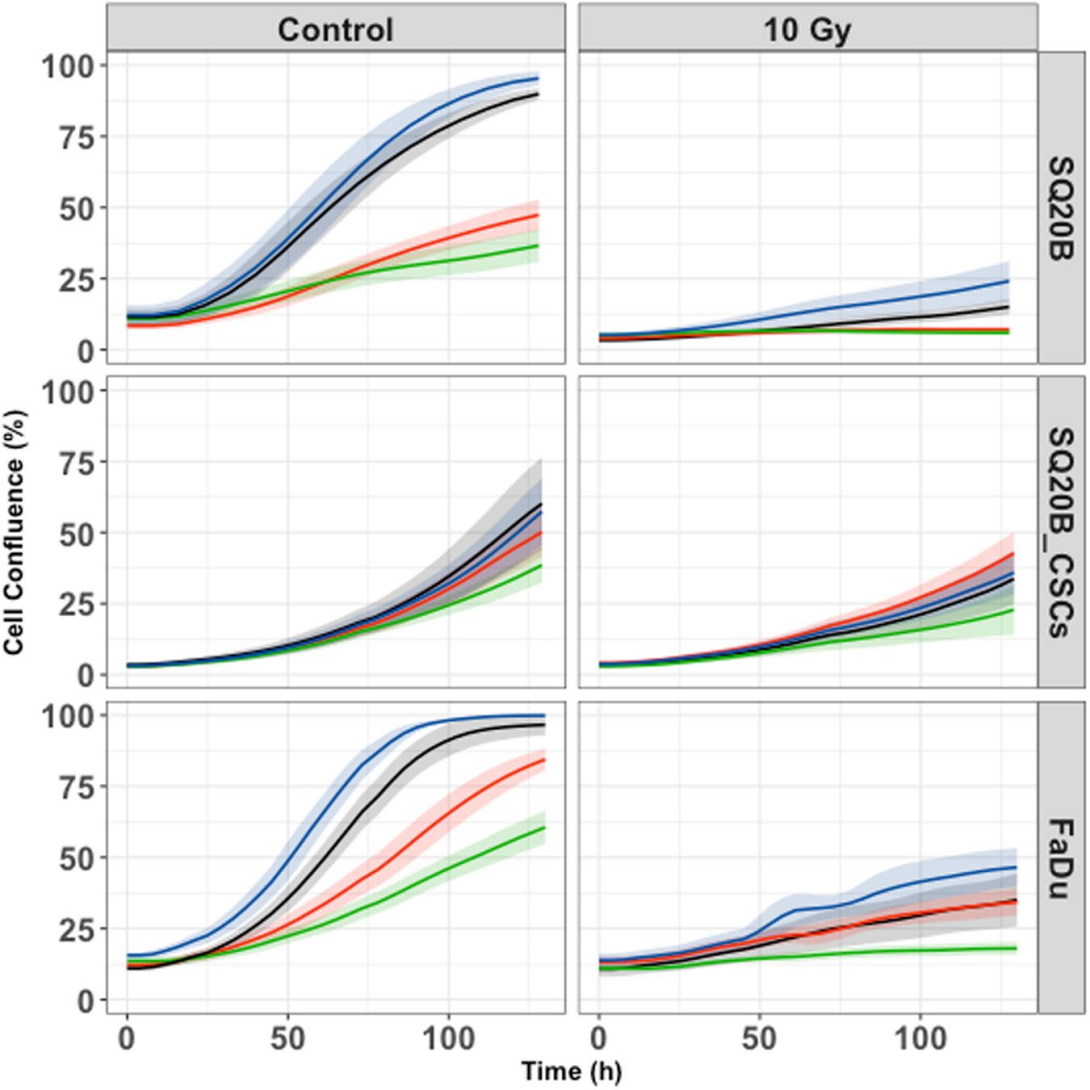
Cell proliferation under each treatment condition, graphically represented by the mean proliferation curves +/− standard deviation. Blue curve, control; black curve, pertuzumab 20 μg/mL; red curve, cetuximab 5 nM; green curve, cetuximab 5 nM + pertuzumab 20 μg/mL. Cell proliferation was measured by an IncuCyte Zoom basic analyser for each cell line under the following treatment conditions: control; cetuximab 5 nM; pertuzumab 20 μg/mL; cetuximab 5 nM + pertuzumab 20 μg/mL; 10 Gy; 10 Gy + cetuximab 5 nM; 10 Gy + pertuzumab 20 μg/mL; 10 Gy + cetuximab 5 nM + pertuzumab 20 μg/mL. Each experiment was performed in triplicate.

Cetuximab significantly inhibited SQ20B (*P* < 0.001) and FaDu (*P* < 0.001) proliferation, whereas it had no effect on SQ20B-CSC proliferation (*p* = 0.8) (Fig. 2 and Table 1). No effect was observed with pertuzumab alone in any cell population. However, the cetuximab–pertuzumab mixture significantly decreased cell proliferation in SQ20B and FaDu populations, and decreased cell proliferation significantly more than cetuximab alone in all three cell populations (*P < *0.001). Applying 10 Gy photon irradiation alone dramatically decreased SQ20B cell proliferation, and decreased SQ20B-CSC proliferation to a lesser degree. Pan-HER blockade with the addition of 10 Gy photon irradiation was more effective than 10 Gy alone in inhibiting the cell proliferation of SQ20B (*P* < 0.001), SQ20B-CSCs (*P* = 0.046) and FaDu (*P* < 0.001).

**Table 1:** Cell proliferation expressed by cell growth rate per hour (Lambda (h-1)) and p-values.

	LAMBDA (h-1) [CI95%]	p-value (Vs Control)	p-value (Vs 10 Gy)	p-value (Vs Cetuximab)
SQ20B				
Control	0.0325 [0.0278–0.037]		—	—
Pertuzumab	0.041 [0.035–0.047]	0.021	—	—
Cetuximab	0.015 [0.013–0.017]	<0.001	—	—
Cetuximab + Pertuzumab	0.00746 [0.006–0.0085]	<0.001	—	<0.001
10 Gy	0.0122 [0.0097–0.147]	—	—	—
10 Gy + Pertuzumab	0.0137 [0.011–0.016]	—	0.42	—
10 Gy + Cetuximab	0.00275 [0.0019–0.0035]	—	<0.001	—
10 Gy + Cetuximab + Pertuzumab	0.000614 [0.0001–0.00012]	—	<0.001	<0.001
SQ20B-CSCs				
Control	0.0351 [0.025–0.045]	—	—	—
Pertuzumab	0.0374 [0.025–0.05]	0.78	—	—
Cetuximab	0.0333 [0.024–0.043]	0.8	—	—
Cetuximab + Pertuzumab	0.0236 [0.017–0.031]	0.061	—	0.047
10 Gy	0.0157 [0.01–0.021]	—	—	—
10 Gy + Pertuzumab	0.0153 [0.01–0.021]	—	0.93	—
10 Gy + Cetuximab	0.014 [0.009–0.019]	—	0.64	—
10 Gy + Cetuximab + Pertuzumab	0.00939 [0.0059–0.013]	—	0.046	0.169
FaDu				
Control	0.036 [0.31–0.40]	—	—	—
Pertuzumab	0.0347 [0.0302–0.0392]	0.75	—	—
Cetuximab	0.0239 [0.0208–0.027]	<0.001	—	—
Cetuximab + Pertuzumab	0.0165 [0.014–0.019]	<0.001	—	<0.001
10 Gy	0.0102 [0.0088–0.012]	—	—	—
10 Gy + Pertuzumab	0.00914 [0.0079–0.014]	—	0.31	—
10 Gy + Cetuximab	0.00715 [0.006–0.008]	—	<0.001	—
10 Gy + Cetuximab + Pertuzumab	0.00377 [0.0031–0.0045]	—	<0.001	<0.001

Leg. *Lambda (h-1): cell growth rate per hour; CI95%: confidence interval; Vs: versus; Gy: Gray*.

### Cetuximab and/or pertuzumab synergized with irradiation to kill HNSCC cells

Isobolographic analyses show that cetuximab–pertuzumab and radiation effects were always synergistic, irrespective of the human cell line concerned (see Supplementary Table S1). By contrast, pertuzumab alone was an antagonist in SQ20B-CSCs and FaDu, as is cetuximab alone in SQ20B-CSCs.

### Cell migration and invasion

**Figure 3:**
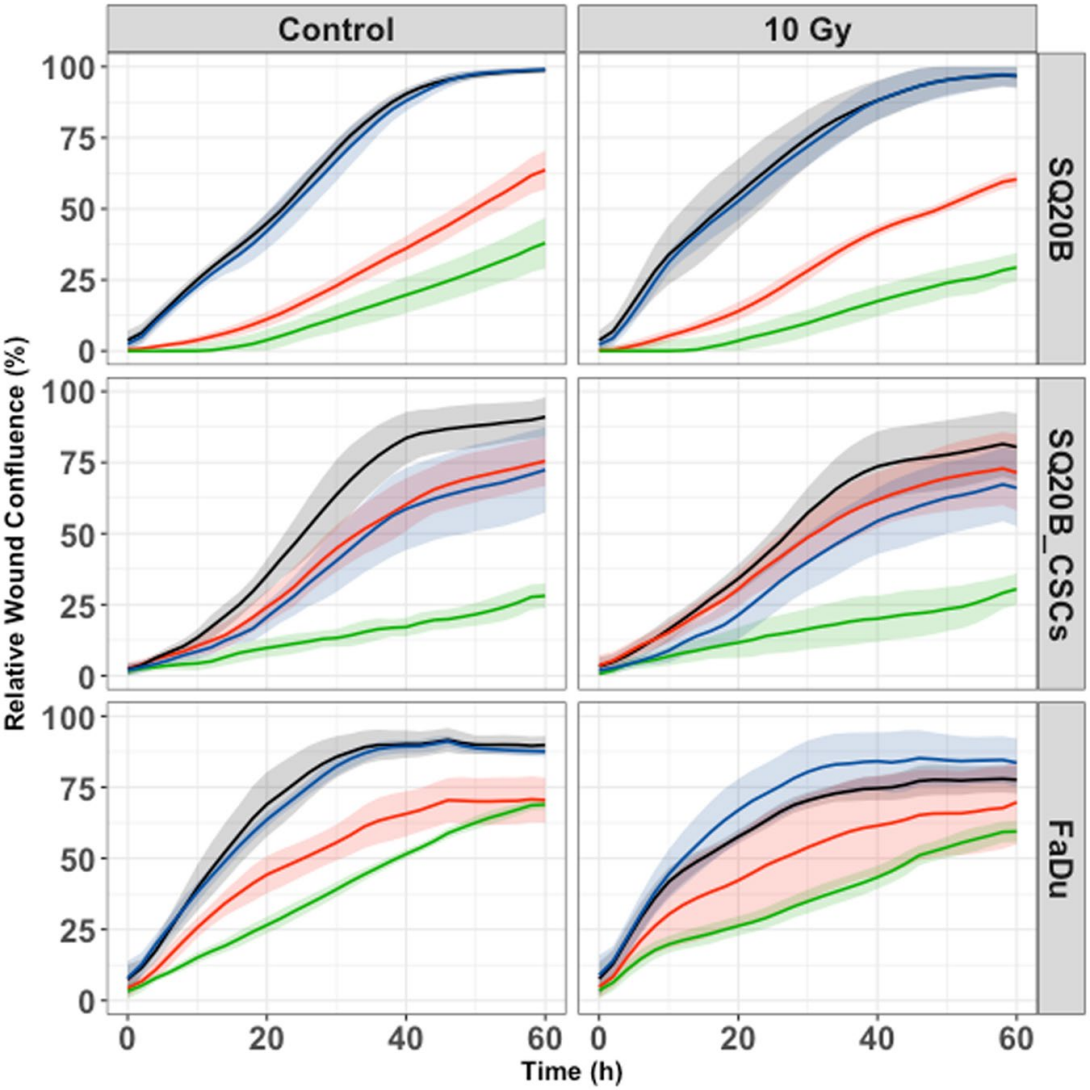
Cell migration under each treatment condition, graphically represented by the mean migration curves +/− standard deviation. Blue curve, control; black curve, pertuzumab 20 μg/mL; red curve, cetuximab 5 nM; green curve, cetuximab 5 nM + pertuzumab 20 μg/mL. Cell migration was measured by an IncuCyte Zoom scratch wound analyser for each cell line under the following treatment conditions: control; cetuximab 5 nM; pertuzumab 20 μg/mL; cetuximab 5 nM + pertuzumab 20 μg/mL; 10 Gy; 10 Gy + cetuximab 5 nM; 10 Gy + pertuzumab 20 μg/mL; 10 Gy + cetuximab 5 nM + pertuzumab 20 μg/mL. Each experiment was performed in triplicate.

**Figure 4:**
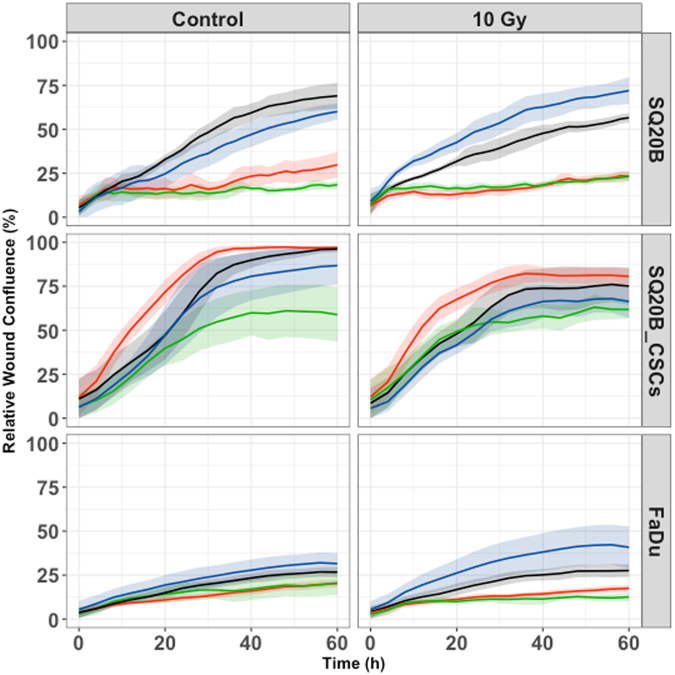
Cell invasion under each treatment condition, graphically represented by the mean invasion curves +/− standard deviation. Blue curve, control; black curve, pertuzumab 20 μg/mL; red curve, cetuximab 5 nM; green curve, cetuximab 5 nM + pertuzumab 20 μg/mL. Cell invasion was measured by an IncuCyte Zoom scratch wound analyser for each cell line under the following treatment conditions: control; cetuximab 5 nM; pertuzumab 20 μg/mL; cetuximab 5 nM + pertuzumab 20 μg/mL; 10 Gy; 10 Gy + cetuximab 5 nM; 10 Gy + pertuzumab 20 μg/mL; 10 Gy + cetuximab 5 nM + pertuzumab 20 μg/mL. Reduced Matrigel (dilution 1/10) was set into each wound. Each experiment was performed in triplicate.

The cetuximab–pertuzumab mixture inhibited both migration and invasion in the three populations (Figs 3 and 4; Tables 2 and 3). The dual treatment decreased SQ20B-CSC migration and invasion more effectively than cetuximab alone (*P < *0.001 and *P* < 0.001) (Figs 3 and 4; Tables 2 and 3). Adding pertuzumab increased effect of cetuximab on migration in all cell populations (SQ20B, *P = *0.0086; SQ20B-CSCs, *P < *0.001; and FaDu, *P* = 0.0491). Alone, 10 Gy photon irradiation appeared to have little effect on cell migration and invasion, as did pertuzumab alone. Furthermore, SQ20B-CSCs invaded and healed the wound faster than SQ20B cells (complete wound healing for SQ20B ≈ 40 hours; complete wound healing for SQ20B-CSCs ≈ 30 hours). SQ20B-CSCs migrated alone, without cell–cell junctions and a mesenchymal phenotype, in contrast to SQ20B cells (Supplementary Fig. S1).

**Table 2:** Cell migration expressed with time to heal 50% of the wound (T50) and p-values.

	T50 (h) [CI95%]	p-value (Vs Control)	p-value (Vs 10 Gy)	p-value (Vs Cetuximab)
SQ20B				
Control	15.5 [12.9–18.1]	—	—	—
Pertuzumab	15.4 [12.8–17.9]	0.94	—	—
Cetuximab	37.8 [31.5–44.1]	<0.001	—	—
Cetuximab + Pertuzumab	52.6 [43.4–61.8]	<0.001	—	0.0086
10 Gy	19.7 [15.8–23.6]	—	—	—
10 Gy + Pertuzumab	17 [13.7–20.3]	—	0.3	—
10 Gy + Cetuximab	43.6 [34.9–52.2]	—	<0.001	—
10 Gy + Cetuximab + Pertuzumab	69.3 [54.4–84.2]	—	<0.001	0.0013
SQ20B-CSCs				
Control	20 [13.3–26.6]	—	—	—
Pertuzumab	23.7 [15.6–31.7]	0.49	—	—
Cetuximab	19.2 [12.7–25.7]	0.86	—	—
Cetuximab + Pertuzumab	54.8 [36.2–73.4]	<0.001	—	<0.001
10 Gy	20.9 [13.8–27.9]	—	—	—
10 Gy + Pertuzumab	31.4 [20.8–41.9]	—	0.093	—
10 Gy + Cetuximab	19 [12.5–24.5]	—	0.7	—
10 Gy + Cetuximab + Pertuzumab	71.1 [45.6–96.6]	—	<0.001	<0.001
FaDu				
Control	16.3 [11.8–20.8]	—	—	—
Pertuzumab	15.8 [11.5–20.1]	0.89	—	—
Cetuximab	31 [22.6–39.4]	0.0011	—	—
Cetuximab + Pertuzumab	46 [33.4–58.5]	<0.001	—	0.0491
10 Gy	17.1 [12.4–21.8]	—	—	—
10 Gy + Pertuzumab	23.2 [16.9–29.5]	—	0.11	—
10 Gy + Cetuximab	31.6 [22.9–40.2]	—	0.0017	—
10 Gy + Cetuximab + Pertuzumab	99.6 [70.2–129]	—	<0.001	<0.001

Leg. *T50 (h): time to heal 50% of the wound in hours; CI95%: confidence interval; Vs: versus; Gy: Gray*.

**Table 3:** Cell invasion expressed with time to heal 50% of the wound (T50) and p-values.

	T50 (h) [CI95%]	p-value (Vs Control)	p-value (Vs 10 Gy)	p-value (Vs Cetuximab)
SQ20B				
Control	29.1 [14.6–43.6]	—	—	—
Pertuzumab	34 [17.1–50.9]	0.67	—	—
Cetuximab	138 [67.4–208.6]	<0.001	—	—
Cetuximab + Pertuzumab	242 [106.7–377.2]	<0.001	—	0.096
10 Gy	33 [16.5–49.5]	—	—	—
10 Gy + Pertuzumab	22.8 [11.4–34.2]	—	0.31	—
10 Gy + Cetuximab	159 [74.7–243.3]	—	<0.001	—
10 Gy + Cetuximab + Pertuzumab	230 [104.6–355.4]	—	<0.001	0.42
SQ20B-CSCs				
Control	19.3 [15.6–23.1]	—	—	—
Pertuzumab	21 [16.9–25.1]	0.53	—	—
Cetuximab	13.8 [11.1–16.5]	0.018	—	—
Cetuximab + Pertuzumab	26.3 [21–31.6]	0.028	—	<0.001
10 Gy	26.1 [19–31.2]	—	—	—
10 Gy + Pertuzumab	27.8 [22.3–33.3]	—	0.66	—
10 Gy + Cetuximab	15.8 [12.5–19.1]	—	<0.001	—
10 Gy + Cetuximab + Pertuzumab	21.3 [16.8–25.8]	—	0.17	0.046
FaDu				
Control	186 [42.9–329]	—	—	—
Pertuzumab	129 [31–227]	0.5	—	—
Cetuximab	520 [88.8–951]	0.071	—	—
Cetuximab + Pertuzumab	652 [103–1200]	0.032	—	0.92
10 Gy	472 [99.6–844]	—	—	—
10 Gy + Pertuzumab	118 [20–216]	—	0.018	—
10 Gy + Cetuximab	1700 [112–3287]	—	0.038	—
10 Gy + Cetuximab + Pertuzumab	5970 [0–12830]	—	<0.001	0.059

Leg. *T50 (h): time to heal 50% of the wound in hours; CI95%: confidence interval; Vs: versus; Gy: Gray*.

### Protein expression

**Figure 5:**
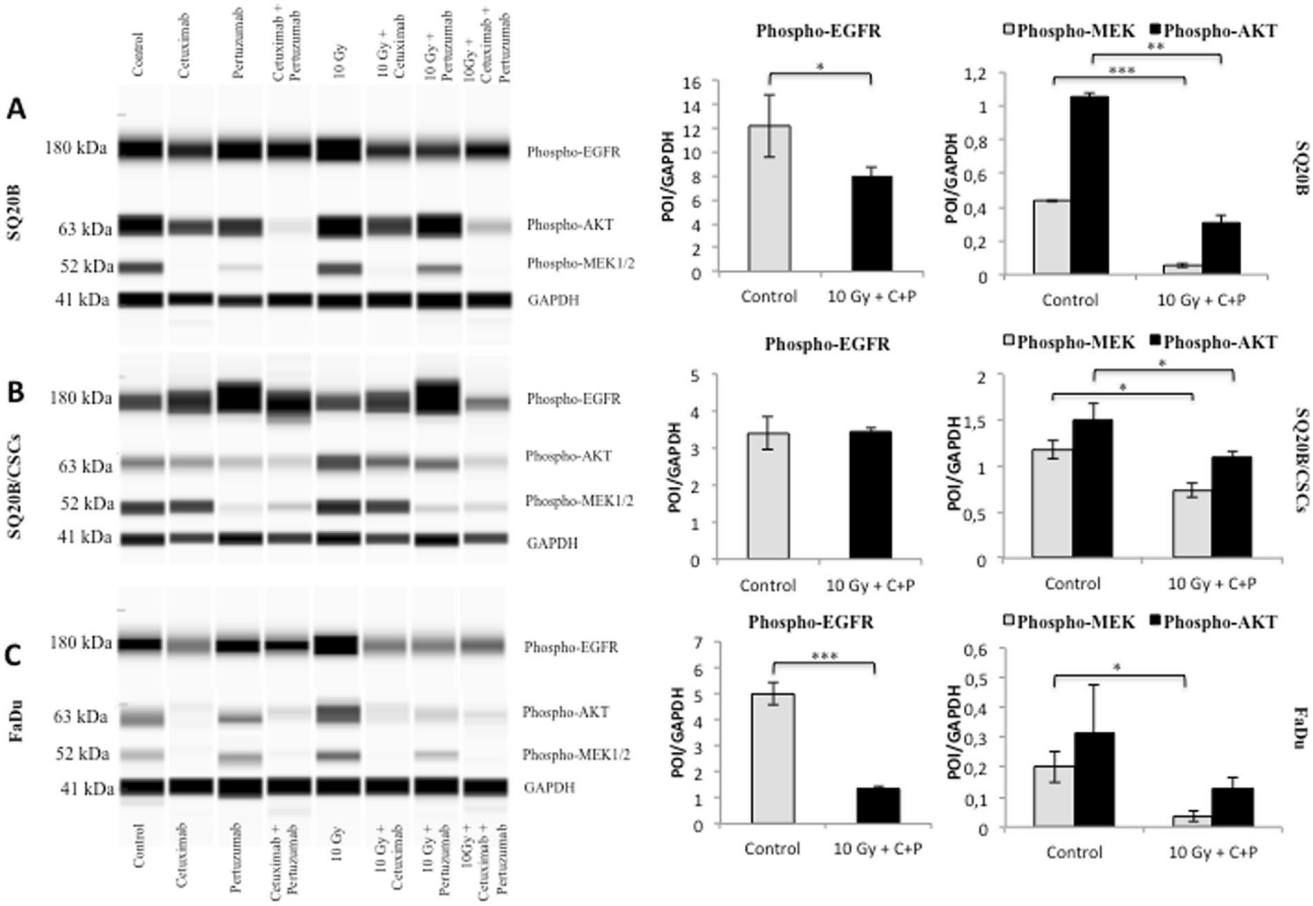
*Treatment impact on protein expression*. The expression of phospho-EGFR (Tyr1068) (182 kDa), phospho-AKT (Ser473) (63 kDa) and phospho-MEK1/2(Ser217/221) (52 kDa) was evaluated under the following treatment conditions: control; cetuximab 5 nM; pertuzumab 20 μg/mL; cetuximab 5 nM + pertuzumab 20 μg/mL; 10 Gy; 10 Gy + cetuximab 5 nM; 10 Gy + pertuzumab 20 μg/mL; 10 Gy + cetuximab 5 nM + pertuzumab 20 μg/mL. Ratios of phospho-EGFR, phospho-AKT and phospho-MEK to GAPDH expression (41 kDa) were calculated and are graphed as (protein-of-interest (POI)/GAPDH). (**A**) SQ20B. (**B**) SQ20B-CSCs. (**C**) FaDu. Statistically significant differences are expressed as *p < 0.05, **p < 0.01 and ***p < 0.001. Each experiment was performed in triplicate.

Phospho-EGFR expression was decreased by cetuximab in SQ20B and FaDu cell lines. Cetuximab–pertuzumab in combination with 10 Gy photon irradiation significantly decreased EGFR activation in SQ20B and FaDu cells (*P* = 0.043 and *P* < 0.001, respectively), but not in SQ20B-CSCs (Fig. 5A–C). The combined treatment with cetuximab–pertuzumab and 10 Gy irradiation significantly decreased phospho-AKT in SQ20B cells (*P* = 0.002), and SQ20B-CSCs (*P* = 0.042). In the same way, the triple treatment significantly decreased Phospho-MEK1/2 in SQ20B cells (*P* < 0.001), SQ20B-CSCs (*P* = 0.041) and FaDu cells (*P* = 0.05). Photon irradiation appeared to increase EGFR phosphorylation in SQ20B and FaDu cells. Interestingly, pertuzumab alone activated EGFR in SQ20B-CSCs. The activation or inactivation of phospho-HER2 and phospho-HER3 did not show any significant differences between the different treatment conditions, and remained extremely low.

## Discussion

The development of resistance to anticancer drugs represents a major obstacle for modern oncology, and understanding cetuximab resistance mechanisms might be the key to improving the outcomes of HNSCC patients. In the present study, we investigated mechanisms of resistance to cetuximab, and demonstrated that HER family members are key central checkpoints. We found that pan-HER blockade significantly inhibited proliferation, invasion and migration in three different HNSCC cell populations.

SQ20B-CSCs were confirmed to be resistant to conventional treatment. Our study confirmed the presence of a highly invasive CSC intrinsic population (SQ20B-CSCs) with mesenchymal characteristics, and which is resistance to concomitant cetuximab plus RT^3,4^. EGFR was highly expressed in SQ20B cells but under-expressed in CSCs, as has been previously demonstrated in HNSCC CSCs^9^, and in another HNSCC population^14^. Interestingly, HER2 and HER3 were expressed at the same level in the three cell populations. These family members are frequently expressed in cell lines studied in the current literature, and might be keys to enhancing therapeutic efficacy against HNSCC^11,12^. Moreover, these receptors are working together, and are even dependent on each other because of their intrinsic characteristics (Fig. 1). In parallel, our cells showed striking differences in their signalling pathways. When SQ20B cells activated the AKT-mTOR pathway (increased phospho-AKT), SQ20B-CSCs activated the RAS-MAPK pathway (increased phospho-MEK1/2). Increasing attention has been paid in recent years to targeting the PI3K-AKT-mTOR pathway, largely with tyrosine kinase inhibitors (TKIs)^15^. Our study revealed that CSCs could escape this pathway by favouring the RAS-MAPK pathway. This observation leads us to think that the upstream extracellular blockade may be more effective than the downstream blockade with TKIs.

We studied here a pan-HER mixture using cetuximab and pertuzumab. Both drugs have been validated clinically. This synergistic combination blocked HER members, their cross-phosphorylation and their downstream signalling. However, our results confirmed that EGFR is the key trigger in HNSCC cell signalling. Pertuzumab alone is inefficient, and seemed to increase EGFR phosphorylation in some cases. By contrast, the combination with cetuximab was synergistic and confirmed the hypothesis of an escape mechanism via HER2 or HER3 coreceptors. In a study published by Wheeler *et al*., cells exposed to cetuximab for weeks developed an overexpression of HER2 and HER3^10^. In fact, HER homo- and heterodimers form a highly redundant group of receptor complexes and thereby add to the complexity of EGFR signalling^16^. Dimerization stimulates HER cytoplasmic kinase activity, leading to auto- and trans-phosphorylation on tyrosine residues, which serve as docking sites for adaptor proteins and enzymes. Survival can be promoted by the constitutive activation of signalling pathways downstream of EGFR, through the mutation or upregulation of other HER family receptors or other receptor tyrosine kinase classes^2^. A high level of activated Akt can occur downstream of the EGFR inhibition through upstream-activated Src, Ras or mutated phosphatase and tensin homolog (PTEN)^17^; amplification of the catalytic subunit of PI3K^18^; or loss of the PTEN tumour suppressor protein^19^. Finally, overexpression of six major HER family ligands can activate HER receptors^20^, resulting in resistance to EGFR inhibition.

Moreover, data suggest that EGFR functionality can also be dependent on its subcellular location^21^. EGFR undergoes translocation into different organelles, where it elicits functions distinctly different from its well-known activity as a plasma membrane-bound receptor tyrosine kinase. Several observations suggest that EGFR can be shuttled into the cell nucleus and mitochondrion upon ligand binding, radiation, EGFR-targeted therapy and other stimuli^22^. Nuclear EGFR behaves as a transcriptional regulator^23^ and seems to be an indicator of poor clinical outcomes. In our study, microscopic observation (Fig. 2E,F) confirmed that the location of EGFR in CSCs is probably intra-cytoplasmic and therefore inaccessible to outer-membranous antibodies. Nuclear EGFR could be a promising therapeutic target according to some studies^24^. Mitochondrial EGFR^25^ also appears to regulate apoptosis, and some authors suggest that EGFR prevents autophagy^26^.

A striking effect of the cetuximab–pertuzumab combination on cell motility was observed. This combination inhibited both migration and invasion in the three cell populations. This inhibition was correlated with down-regulation of both AKT-mTor and Ras-MAPK signalling (Fig. 5). Targeting these downstream signalling pathways is of major interest in HNSCC^15^. It has been suggested that EGFR can induce epithelial–mesenchymal transition-like changes, leading to invasion and migration through action on the extracellular matrix^27^. Our study demonstrated that HER2 and HER3 are also involved in cell motility. Data from other studies suggest that radiation enhances invasion/migration. This effect was not observed in our study, because of the type of experiment (scratch wound) and irradiation doses. A limit of this therapeutic combination is, of course, treatment tolerance, as the treatment has been shown to be toxic in a study of colon cancer patients^28^. These results should be extrapolated to the clinic with caution, knowing that *in vitro* conditions only partially represent clinical reality^29^. However, preliminary work on dose reduction indicated that the therapeutic effectiveness of the dual treatment could be maintained after reducing the dose of cetuximab by 50% (Supplementary Data Fig. S2), thereby reducing toxicity to a manageable level.

To conclude, this study shows that conventional treatments, such as cetuximab with concomitant RT, are missing a cell-resistant CSC subpopulation. It also demonstrates that better knowledge of the mechanisms of cellular resistance in HNSCC could lead us to propose new drug-combinations in association with photon radiation to increase therapeutic efficacy. If recent technological developments in modern RT improve the efficacy/tolerance ratio, antibody combinations targeting the entire HER family in association with photon radiation may become major weapons for reversing cancer resistance.

## Methods

### Cell culture

The HNSCC SQ20B cell line was derived from a recurrent laryngeal cancer (John Little, USA). This cell line is p53-mutated and Human Papilloma Virus-negative. The HNSCC FaDu cell line (obtained from the American Type Culture Collection [ATCC], USA) was derived from an oropharyngeal cancer, and used in parallel with SQ20B cells. CSCs (SQ20B-CSCs) were obtained as previously described^30,31^.

### Irradiation

Photon irradiation was performed with an X-RAD320 irradiator (Precision X-ray Inc., North Branford, CT, USA) at the Faculty of Medicine in Lyon Sud of the Université Lyon 1 (UMS2444/US8 platform, France), at a dose rate of 2 Gy/min. The total irradiation dose was 10 Gy for proliferation, migration and invasion assays, and for protein expression analysis.

### Drugs

Cetuximab (C-225, Merck Serono, Darmstadt, Germany) and pertuzumab (Roche SAS, Boulogne-Billancourt, France) were provided by the Pharmaceutical Department of the Centre Hospitalier Universitaire Lyon-Sud (Pierre Bénite, France). Cetuximab was used at a 5 nM concentration, as previously described^9^. Pertuzumab was used at a 20 μg/mL concentration, following published methods^32^. Cells were treated with cetuximab and/or pertuzumab 1 h before 10 Gy irradiation.

### Microscopy

Phalloidin staining was performed to study actin, and EGFR was stained to study its cellular localization. Briefly, 2 × 10^5^ cells were seeded in a six-well plate on slats and permeabilized with 4% paraformaldehyde, then blocked with a 10% PBS–foetal bovine serum solution. For phalloidin staining, cells were incubated in a 1% PBS–BSA solution with 1/5000 DAPI (Sigma-Aldrich, Saint-Louis, MO, USA) and 1/200 phalloidin-fluorescein antibody (Sigma-Aldrich). For EGFR staining, cells were incubated in 0.1% PBS–Triton solution for 2 h with 1/100 EGFR primary antibody (sc-03; Santa Cruz Biotechnology, Santa Cruz, CA, USA). Then cells were incubated in a 0.1% PBS–Triton solution for one hour with 1/200 anti-rabbit FITC secondary antibody (Santa Cruz Biotechnology, Santa Cruz, CA, USA) and 1/5000 DAPI. Sections were mounted using glass coverslips and mounting medium (Sigma-Aldrich), and then visualized on a Zeiss fluorescence microscope (AxioImager Z2, Zeiss, Oberkochen, Germany).

### Cell proliferation

The IncuCyte ZOOM^®^ live cell imaging system (Essen BioScience, Ann Arbor, MI, USA) was used to measure cell proliferation. Cells (5 × 10^4^) were plated in 96-well plates for 16 h. Each well was treated according to different conditions (control, 5 nM cetuximab; 20 μg/mL pertuzumab; 5 nM cetuximab + 20 μg/mL pertuzumab) and irradiated at 10 Gy for 1 h after treatment. Growth curves (proliferation) were built from confluence measurements acquired during round-the-clock kinetic imaging (one picture every two hours). The cell confluence was measured for 120 h. Each experiment was performed in triplicate.

### Migration and invasion assays

For migration and invasion assays, cells were plated in 96-well ImageLock plates (Essen BioScience). SQ20B and FaDu cells (4 × 10^5^) and SQ20B-CSCs (3.5 × 10^5^) were plated for 16 h in order to achieve 90–100% confluence. Then the plates were scratched with a 96-well WoundMaker™ (Essen BioScience). For the invasion assays, 50 μL of reduced Matrigel (dilution 1/10; BD Biosciences, Franklin Lakes, NJ, USA) was added to each well. Migration/invasion was detected by IncuCyte scanning one image per well, every 2 h, for 30 h for migration and 40 h for invasion. The time course of cell migration/invasion was quantified using IncuCyte ZOOM software, measuring the relative wound density (as a percentage) for each condition over time. Each experiment was performed in triplicate.

### Protein expression levels

Cell pellets were lysed in 50 mM Tris buffer (pH 8.0), 150 mM NaCl, 1% Triton X-100, protease inhibitors (Complete Mini, Roche) and anti-phosphatases (PhosSTOP, Roche) for 1 h at 4 °C. Lysates were centrifuged for 20 min at 15000 × *g* at 4 °C. Protein expression studies were performed by WES, an automated capillary-based size sorting system (ProteinSimple, San Jose, CA, USA)^33,34^. Data were analyzed using Compass software (ProteinSimple, San Jose, CA, USA). The primary antibodies used were EGFR (sc-03; Santa Cruz Biotechnology, Santa Cruz, CA, USA), phospho-EGFR (Tyr1068; Cell Signaling Technology, Danvers, MA, USA), HER2 (Cell Signaling Technology), HER3 (Cell Signaling Technology), phospho-HER2 (Y1221/1222) (Cell Signaling Technology), phospho-HER3 (Y1283) (Cell Signaling Technology), phospho-AKT (Ser473; Cell Signaling Technology) and phospho-MEK1/2 (Ser217/221; Cell Signaling Technology) at a 1/50 dilution, and GAPDH (Santa Cruz Biotechnology) was used as a reference. Protein expressions are represented as digitized images of blotting by quantitative chemiluminescence. Each experiment was performed in triplicate.

### Cell Cycle analysis

Cells (1 × 10^6^) were plated in 25 cm² BD Falcon flasks (BD Falcon, Franflin Lakes, NJ, USA) for 16 h. Cells were treated according to the drug combinations specified previously, and irradiated at 10 Gy for 1 h after treatment. The cells were then harvested, centrifuged, washed once with PBS and fixed in cold 70% ethanol for at least 24 h at −20 °C. Fixed cells were centrifuged at 300 × *g* for 5 min and resuspended in PBS. Cells were then incubated for 30 min at room temperature in the dark in a 1/2000 DAPI solution. Finally, flow cytometry was conducted (using a BD FACSAria III, BD Biosciences) and the results were analysed using BD FACSDiva Software (BD Biosciences) to determine the relative DNA content. The cell cycle distribution was calculated after appropriate gating of the cell population. The results of this analysis are available in the supplementary data.

### Statistical analysis

Analysis of proliferation, migration and invasion data was carried out using a non-linear mixed-effects model to take into account the longitudinal nature of the data and the potential inter-experiment variability^35^. Proliferation data were analyzed using a generalized logistic growth model:

$$\{\begin{array}{c}\frac{dCC}{dt}=\lambda \times CC\times (1-C{C}^{\alpha })\\ CC(t=0)=C{C}_{0}\end{array}$$

where *CC*(*t*) is the cell confluence expressed as a percentage at time *t*, and *CC*_0_ is its initial value at *t* = 0 h. The parameters *λ*(h^−1^) and α correspond respectively to the growth rate and the curvature. Migration and invasion data were analyzed using a generalized Hill equation:

$$WC(t)=\frac{{t}^{\alpha }}{{T}_{50}^{\alpha }+{t}^{\alpha }}$$

where *WC* is the wound confluence at time *t*. The parameters and *T*_50_ correspond respectively to the curvature and the time at which 50% of the wound was recovered. The influence of the different treatment conditions on for proliferation data and *T*_50_ for migration and invasion data was tested. Statistical differences were assessed using likelihood ratio tests^36^. Analyses were done with Monolix^**®**^ software (Lixsoft-Incuballiance, Paris, France).

The differences between protein expressions (protein-of-interest/GAPDH) were determined by a t-test. The minimum level of significance was set at *p* < 0.05.

Dose–response interactions between radiation (photon therapy) and cetuximab and/or pertuzumab were evaluated using the classical isobolographic method described by Steel and Peckham^37^. The theoretical basis and procedure for the isobologram method have been described in detail^38^. The coordinates of the experimental point are the cetuximab and/or pertuzumab concentration and the radiation dose, which, when combined, give the level of efficacy. For a given level of efficacy, an “envelope of additivity” curve was calculated from the dose–effect curves of cetuximab and/or pertuzumab combined with irradiation (one dose of 10 Gy) and from the dose–effect curves of radiation alone (one dose of 10 Gy). If the experimental point falls above, beyond or under the limits of the “envelope of additivity”, cetuximab and/or pertuzumab and radiation in combination give rise to antagonistic, additive or synergistic effects, respectively.

### Availability of data and materials

All data generated or analysed during this study are included in this published article.

## Electronic supplementary material

Supplementary Information
