# Supplementary material for: Dual “mAb” HER family blockade in head and neck cancer human cell lines combined with photon therapy
Source: Sci Rep. 2017 Sep 22;7:12207. doi: 10.1038/s41598-017-12367-7 (PMC5610257; doi:10.1038/s41598-017-12367-7)
Supplement: Supplementary file 1 — Supplementary Information [file 41598_2017_12367_MOESM1_ESM.doc]

**Supplementary Data**

**Dual “mAb” HER family blockade in head and neck cancer human cell lines combined with photon therapy.**

Jean-Baptiste Guy, MD, PhD Student,^1,2^, Benoîte Méry, MD, PhD Student,^1,3^, Edouard Ollier, MD,^4^ Sophie Espenel, MD,^1,2^, Alexis Vallard, MD,^2^ Anne-Sophie Wozny, PharmD, PhD Student,^1,5^ Stéphanie Simonet, PhD Student,^1^ Alexandra Lauret, PhD Student,^1^ Priscillia Battiston-Montagne, BSc,^1^ Dominique Ardail, PhD,^1,5^ Gersende Alphonse, PhD,^1^ Chloé Rancoule, PhD,^2^, Claire Rodriguez-Lafrasse, PharmD PhD,^1,5,*^ Nicolas Magné, MD, PhD,^1,2,*^

1. Université Lyon 1, UMR CNRS 5822 /IN2P3, IPNL, PRISME, Laboratoire de Radiobiologie Cellulaire et Moléculaire, Faculté de Médecine Lyon-Sud, F-69921 Oullins cedex, France

2. Département de Radiothérapie, Institut de Cancérologie de la Loire - Lucien Neuwirth, 42270 St Priest en Jarez, France

3. Département d’Oncologie Médicale, Institut de Cancérologie de la Loire - Lucien Neuwirth, 42270 St Priest en Jarez, France

4. Dysfonction Vasculaire et Hémostase, INSERM, U1059, 42270 Saint-Priest en Jarez, France

5. Hospices Civils de Lyon, Centre Hospitalier Lyon-Sud, 69495 Pierre-Bénite, France

* *Authors contributed equally to this work and should be considered as joint last author*

**Correspondence**

Pr Nicolas Magné

Laboratoire de Radiobiologie Cellulaire et Moléculaire

Faculté de Médecine Lyon Sud

165 Chemin du Grand Revoyet – BP 12

69921 OULLINS Cedex

Tel: +33 4 26 23 59 58

Fax: +33 4 26 23 59 66

[nicolas.magne@icloire.fr](mailto:nicolas.magne@icloire.fr)

**Supplementary Table S1: *Isobolographic analysis at 100 h and 140 h of the three human cell lines (SQ20B; SQ20B-CSCs; FaDu) with cetuximab and/or pertuzumab and one dose of ionizing irradiation with photons (10 Gy)*.**

cetuximab pertuzumab cetuximab/pertuzumab

Human cell Lines

SQ20B Synergistic Additive Synergistic

SQ20B/CSCs Antagonist Antagonist Synergistic

FaDu Additive Antagonist Synergistic

**Supplementary Figure S1:** ***Microscopic observation (10×) of the scratch wound experiment.***

**A.** SQ20B cells healing the wound at times 0, 15, 30 h. **B.** Wound-healing of SQ20B-CSCs at times 0, 12, 24 h.

**Supplementary Figure S2: *Cell proliferation curves with a modified treatment dose.* *Blue curve, control; red curve, cetuximab 5 nM; green curve, cetuximab 2.5 nM (50% dose) + pertuzumab 20μg/mL (100% dose).***

Cell proliferation was measured by an IncuCyte Zoom basic analyser for each cell line exposed to the following treatment conditions: control; cetuximab 5 nM; cetuximab 2.5 nM (50% dose) + pertuzumab 20 μg/mL (100% dose).

**Supplementary Figure S3: *Cell cycle analysis for SQ20B cells exposed to each treatment condition.***

Sub-G1 and G2/M phases are represented in histograms showing the percentage of cells in each phase. Treatment conditions: control; cetuximab 5 nM; pertuzumab 20 μg/mL; cetuximab 5 nM + pertuzumab 20 μg/mL; 10 Gy; 10 Gy + cetuximab 5 nM; 10 Gy + pertuzumab 20 μg/mL; 10 Gy + cetuximab 5 nM + pertuzumab 20 μg/mL.
